# DNA sequencing at the picogram level to investigate life on Mars and Earth

**DOI:** 10.1038/s41598-023-42170-6

**Published:** 2023-09-15

**Authors:** Jyothi Basapathi Raghavendra, Maria-Paz Zorzano, Deepak Kumaresan, Javier Martin-Torres

**Affiliations:** 1 Department of Planetary Sciences, School of Geosciences, University of Aberdeen, Meston Building, Aberdeen, AB24 3UE Scotland; 2 Centro de Astrobiología (CAB), CSIC-INTA, 28850 Torrejón de Ardoz, Madrid Spain; 3 School of Biological Sciences, Queen’s University Belfast (QUB), Belfast, BT9 5DL Northern Ireland; 4 Instituto Andaluz de Ciencias de la Tierra (CSIC-UGR), 18100 Granada, Spain

**Keywords:** Microbiology, Environmental sciences, Planetary science

## Abstract

DNA is an incontrovertible biosignature whose sequencing aids in species identification, genome functionality, and evolutionary relationships. To study life within the rocks of Earth and Mars, we demonstrate, in an ISO5 clean room, a procedure based on nanopore technology that correctly identifies organisms at picogram levels of DNA without amplification. Our study with *E. coli* and *S. cerevisiae* DNA samples showed that MinION sequencer (Oxford Nanopore Technologies) can unequivocally detect and characterise microbes with as little as 2 pg of input with just 50 active nanopores. This result is an excellent advancement in sensitivity, immediately applicable to investigating low biomass samples. This value is also at the level of possible background contamination associated with the reagents and the environment. Cultivation of natural and heat-treated Martian analogue (MMS-2) regolith samples, exposed to atmospheric water vapour or in increasing water concentrations, led to the extraction of 600–1000 pg of DNA from 500 mg of soil. Applying the low detectability technology enabled through MinION sequencer for a natural low biomass setting, we characterised the dry MMS-2 and found few soil-related organisms and airborne contaminants. The picogram detection level and the procedure presented here, may be of interest for the future Mars sample Return program, and the life research and planetary protection studies that will be implemented through the sample safety assessment.

## Introduction

Investigating active life forms in extremely low biomass environments is a topic of interest for expanding our knowledge of Earth’s biodiversity and the search for life on Mars^1, 2^. Deoxyribose Nucleic Acid (DNA) is a functional biopolymer that contains all the biological instructions (genetic information) that make each species unique and can help delineate taxa^3^. However, due to its low abundance in specific environments, such as rocks, one of the biggest challenges for investigating the microbiome of soils and rocks is the ability to extract it efficiently and characterise it without any amplification. Polymerase chain reaction (PCR) is highly sensitive to contamination and DNA polymerases are prone to error potentially leading to mutations in PCR products that can produce ambiguous results^4^. Non-PCR-based DNA amplification techniques, such as Multiple Displacement Amplification (MDA) with a low error frequency^5^, also exhibit a few limitations such as primer-primer interactions and over-amplification of alleles (preferential amplification). Hence, for the study of low concentrations of DNA, there is a need for new technologies with improved efficiency, sensitivity, and specificity.

The Mars Sample Return (MSR) mission will bring to Earth by 2033 a collection of solid samples (rock, regolith and aeolian dust) acquired by Perseverance rover from the top 5 cm of the Martian surface^6, 7^. Since 2021, the rover has collected samples, while it explores and characterises the environment of Jezero crater^8–15^. At the end of this mission, once on Earth, these samples will be investigated in depth within terrestrial laboratories to cover a variety of scientific objectives^16^.The detection of organic molecules in Gale and Jezero craters^13, 17–22^, in tandem with favourable environmental conditions around 3.5 billion years ago, indicate that all the ingredients necessary for life as we know it were likely present on Mars, making it a possible habitable site. However, it is still unknown if these organics were produced biologically or abiotically and, because of the limitations of the instrumentation type used in these missions, in-situ exploration missions cannot assess by themselves if there are extinct or extant life form, within the Martian surface^23^. Life on Mars can only be proven on Earth by analysing with highly sensitive instrumentation, the Mars Sample Return collection within a clean room environment. As these samples come from a potentially habitable planet, contamination control in the environment where the samples are manipulated will be as important as the search for life itself. In addition, and for planetary protection purposes, the search for potentially replicating life will be part of the sample safety assessment protocol^24^ that will be applied to the sample collection within the Bio-Safety Level 4 (BSL 4) laboratory environment, in containment^25–27^.

Each of the Martian samples that will be brought to Earth, will contain about 15 g of rock, aeolian dust or regolith (regolith on Mars is the unconsolidated, loose, heterogeneous superficial collection of fragmented rock, this material can be coarse, or fine, or an incredibly fine powder-like dust) encapsulated with a headspace gas of Martian atmosphere. It has been proposed that hundreds of milligrams to a few grams per Martian sample type should be used exclusively to inform the initial biohazard and sample safety assessment studies^24^. However, considering the low biomass concentration in Martian analogue environments, which is between ~ 10^3^ and 10^5^ cells/g and the average bacterial genome size, ranging from ~ 0.5–20 fg (0.5–20 10^–15^ g) of DNA per genome^28–30^, we can extrapolate that any life-detection experiment should meet the requirement to detect at most 0.5 × 10^–12^ to 20 × 10^–10^ g of DNA per g of rock (i.e., 0.0005 ppb to 2 ppb levels in mass ratio). Recent studies in the Atacama Desert, one of the best Martian analogue environments due to its extreme aridity, confirm some of these upper bound ranges of low levels of biomass in rock samples^23^, getting to, at most, 1 μg of DNA per gram of soil (1 ppm). In preparation for this MSR research, in this work, we investigate the sensitivity of MinION sequencer to detect extremely low concentrations of DNA, that could also be present in a regolith sample or a crushed rock at the level of picogram (ppb to ppm level concentrations in mass).

The purpose of this work is to define new limits of concentration for DNA sequencing that could also be applicable in diverse research areas. In this research, it is assumed that if there is a living organism within the returned Mars Sample Collection with the possibility to replicate (and thus, the type of organism that background planetary protection protocols need to contain and control), it relies on the same chemical processes as terrestrial organisms and it codes its genetic information with the known bases (ATGC for DNA, and AUGC for RNA) that are ubiquitously used by life on Earth. Without loss of generality, we will focus on DNA, using DNA-specific soil extraction reagents as RNA is not stable, making its recovery from soil and sediment samples challenging^31^ and thus we focus first on DNA. We are benchmarking our calibration studies against DNA known masses of the order of a few pg (10^−12^ g).

## Methods

All the extractions, dilutions and library preparation for sequencing were carried out inside the clean room environment of ISO (International Organization for Standardization) level 5 (ECSS-Q-ST-70-55C standard)^32^. An ISO 5 Class clean room is a semi-closed ultra clean environment that utilises High Efficiently Particle Air (HEPA) filtration systems to maintain air cleanliness levels of a maximum of 100 particles (≥ 0.5 µm) per cubic meter of the inside air.

### Test samples

*Escherichia coli* (NCTC 9001 Lenticule disc, Sigma Aldrich, UK) with an average genomic size of ~ 5 Mb and yeast from *Saccharomyces cerevisiae*, Type II (YSC-2, 51,475, Sigma Aldrich) with an average genomic size of ~ 12 Mb were chosen as a source of single bacterial and fungal DNA, respectively. We used the above representatives to test if the significant difference in genomic size or the molecular weight of the DNA plays any role in low detection. The bacterial discs or yeast powder were dissolved in sterile 1X PBS (4 g in 10 ml), and the DNA was extracted using DNeasy Powerlyzer powersoil kit (12,855, Qiagen, UK) according to the manufacturer’s instructions. The DNA yield ranged from 2 to 10 ng/µl, which was serially diluted to 1 ng/µl and 0.1 ng/µl using nuclease-free water (NEB, UK) and quantified using 1X dsDNA High Sensitivity (HS) assay kit in Qubit® 4.0 fluorometer (Q33230, ThermoScientific) and Nanodrop One spectrophotometer (for the extractions). Our single species sample tests included DNA amounts starting with 1000 pg (1 ng) and continuing to 750 pg, 500 pg, 250 pg, 100 pg, 50 pg and 10 pg. The Qubit 4.0. fluorometer sensitivity is limited to 10 pg/µl. For concentrations lower than 10 pg/µl, the operator cannot accurately quantify the amount of DNA. Separate MinION flongle flowcells (R10.4.1) were used for each test. For testing a mixed community, ZymoBIOMICS microbial community DNA standard was used (D6305, Cambridge Bioscience, UK). Zymobiomics offers a mock community DNA standard (20 ng/µl, Table 1), containing eight bacterial species and two yeasts. Lower concentrations of the mock community DNA were obtained by serially diluting the stock DNA in nuclease- free water (NEB, UK) and quantified using Qubit® 4.0. fluorometer.

**Table 1:** The ZymoBIOMICS® defined Microbial Community Standard with gDNA abundance. Approximate amount of DNA that could be present in 100 pg of diluted sample.

Microorganism	gDNA abundance (%)	DNA proportion in 100 pg
*Pseudomonas aeruginosa*	12	12 pg
*Escherichia coli*	12	12 pg
*Salmonella enterica*	12	12 pg
*Lactobacillus fermentum*	12	12 pg
*Enterococcus faecalis*	12	12 pg
*Staphylococcus aureus*	12	12 pg
*Listeria monocytogenes*	12	12 pg
*Bacillus subtilis*	12	12 pg
*Saccharomyces cerevisiae*	2	2 pg
*Cryptococcus neoformans*	2	2 pg

### Incubation experiments with Mojave Mars Simulant-2 (MMS-2):

**Figure 1:**
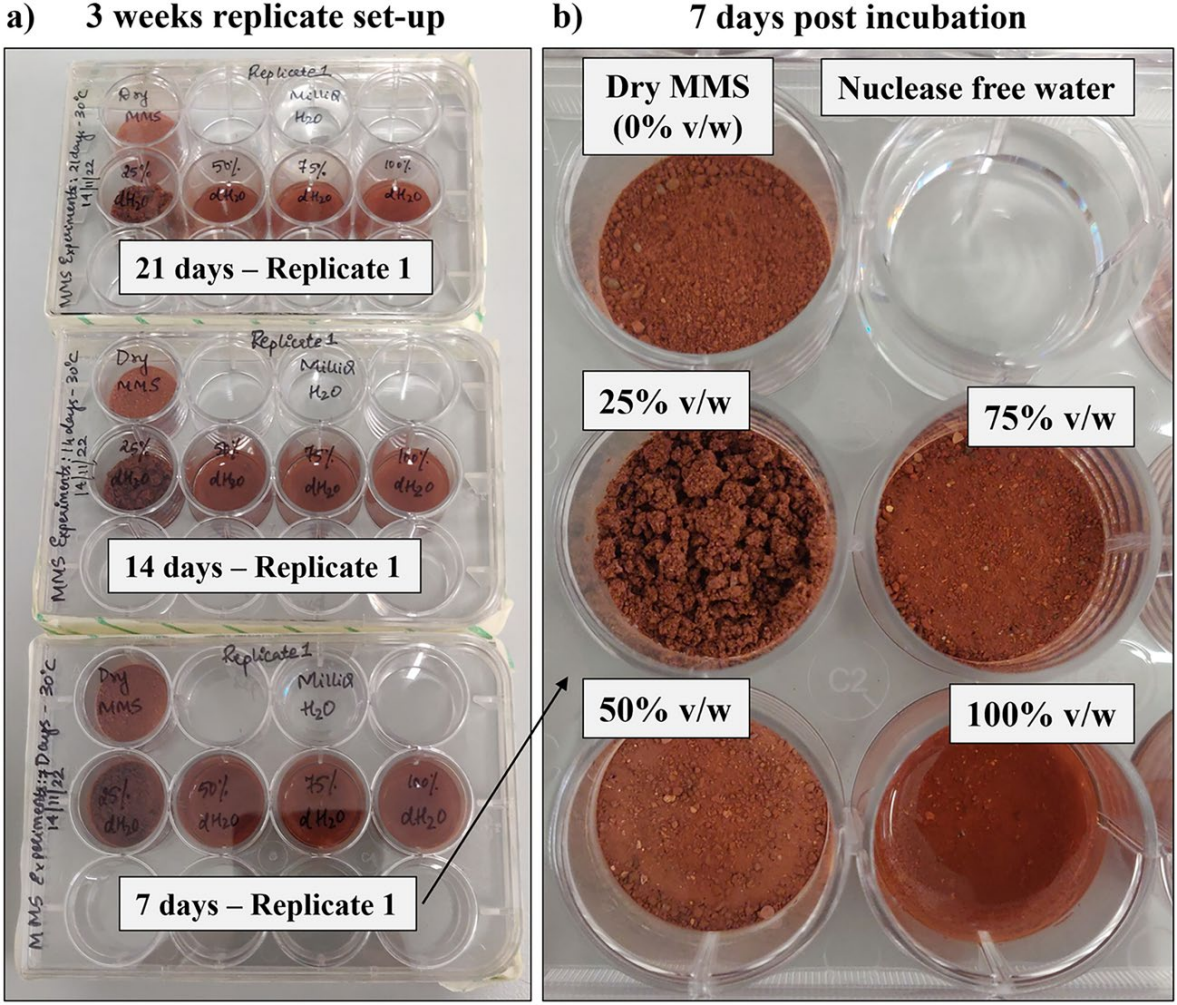
MMS-2 culturing setup (**a**) The three different 12-well plates were incubated during three incubation periods. Each was opened for study at the end of 7 days, 14 days, or 21 days period, and then discarded to prevent recontamination. (**b**) Close view of the soil experiment after 7 days of incubation at 30 °C. The only difference among the soil experiments was the amount of added water, none for the dry MMS case, and 250 µl, 500 µl, 750 µl and 1000 µl for the 25%, 50%, 75% and 100% v/w wells, respectively. A well was filled with only nuclease free water for control. The humidity in each well was monitored with a temperature and RH iButton sensor in a similar setup (not shown). Area of each well: ~ 3.8 cm^2^.

MMS-2 is a super fine grade processed basalt rich in iron III oxide, magnesium oxide, sulfates and silicates, acting as an excellent geologic analogue to the surface of Mars^33^. The natural MMS-2 (Martian Garden, Austin, Texas) soil was weighed and transferred into a 12-well sterile plate (Corning™ Costar™ TC-treated). Five wells were filled with 1 g each, and one was filled with MilliQ water as a control. In order to monitor the microbial growth rate with respect to water availability, only one well was left dry, and MilliQ water was added to the other wells in different concentrations of 250 µl, 500 µl, 750 µl and 1000 µl respectively. After the addition of water, the wells were homogenised with a sterile tip. The 12-well plates were closed with an autoclave tape and incubated at 30 °C. Four incubation periods were chosen i.e., 0, 7, 14 and 21 days (Fig. 1a). Three technical replicates for each condition were used in parallel for DNA extractions. To prevent evaporation of the negative control well, we started with 3 ml of nuclease-free water to have a minimum of 500 µl left for extraction by the end of 21 days incubation period. We observed that, by the end of 7th day of incubation at 30 °C, some of the water from both control and soil wells were evaporated (Fig. 1b). To avoid contamination, each replicate was opened for analysis at the end of its designed time point and then discarded.

For an additional control case, we pre-treated a batch of MMS-2 with a heat- shock by heating the MMS-2 regolith to 125° C for 10 h in a hot conventional air oven (Model OHG050 XX2.5, GallenKamp). Note that these high temperatures are usually applied to sterilise materials, in a procedure called Dry Heat Microbial Reduction method (DHMR)^34^, which is applied in space missions for planetary protection purposes. This process dehydrates the soil and should presumably kill most microorganisms, reducing the bioburden by orders of magnitude. The MMS-2 was filled in a sterile steel petri dish with a lid and was uniformly covered with aluminium foil before placing it in the hot air oven. After cooling the soil, the petri dish was opened inside the clean room before setting up the experiments.

### Water activity monitorisation

The relative humidity (RH) and temperature were measured for 21 days, every 4 h, using the Maxim Integrated iButtons (DS1923-F5, Digi-Key, UK) data loggers and the software OneViewWire (64bit, version 0.3.19.47). For data logging, two analogue experiments to the ones of Fig. 1a (one for natural MMS-2 and one for dry heated MMS-2) were executed and only opened at the end. The iButtons were taped on the inner part of the lid using double-sided tape, such that the sensor membrane is facing inside the well for readings.

### DNA extraction from MMS-2

**Figure 2:**
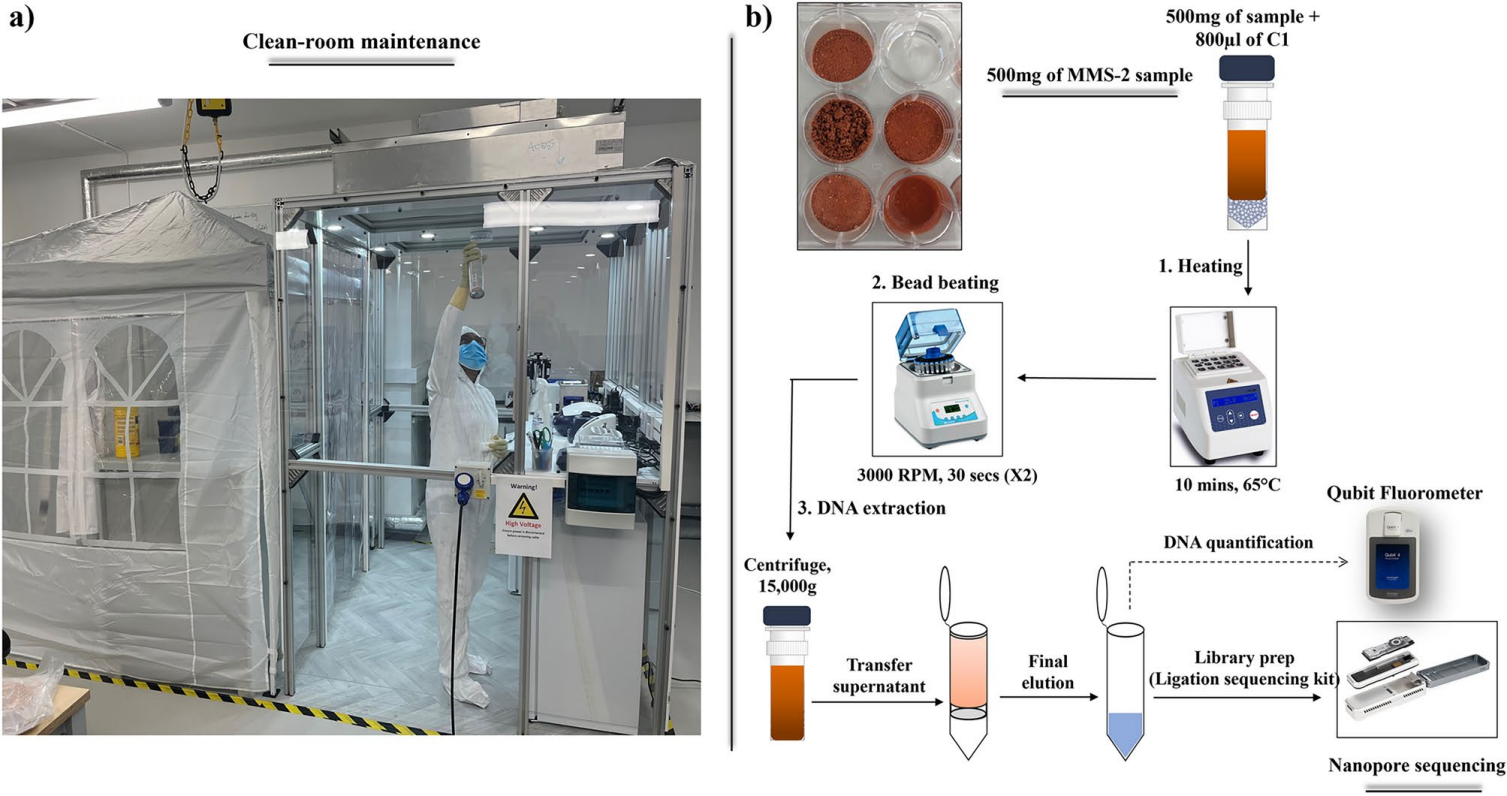
Schematic representation of the end-to-end procedure. (**a**) ISO-5 clean room maintenance: Before any experimental setup, the working table, inside wall panels and equipment were sprayed with 70% isopropyl alcohol and Chemgene HLD_4_L. The space was thoroughly wiped using sterile cleanroom wipes. (**b**) Graphical representation of the DNA extraction and analysis procedure, from 500 mg of MMS-2 sample. The obtained DNA can be directly fed to the nanopore sequencing instrument or, alternatively, be used for DNA mass quantification if the concentration is above 10 pg/µl.

DNeasy powersoil pro kit (47014, Qiagen, UK) was used for all the extractions. 500 mg of the soil from the incubated wells were aseptically transferred by weighing directly into the Powerbead tubes. The remaining 500 mg were stored as a backup at -20 °C for future analysis if needed. As a negative control, 500 µl of the incubated MilliQ water without soil was added separately into the Powerbead tube. The tubes were then filled with 800 µl of the C1 lysis buffer and were briefly vortexed and incubated at 65 °C for 10 min. The Powerbead tubes were then secured to a bead beater (BeadBug™ Microtube homogeniser) and homogenised at 3000 RPM, for 2 cycles, 30 s each, with a 30 s break between each cycle. The tubes were centrifuged at 15,000 g for 2 min and 400–500 µl of the supernatant was carefully transferred to a centrifuge tube using sterile 100–200 µl filter tips, for maximum recovery. Hereafter, the extraction procedure followed the manufacturer's instructions, and the final DNA was eluted in 30 µl of nuclease-free water. The DNA was quantified using Qubit 1X High Sensitivity (HS) assay kit for Qubit® 4.0 fluorometer (ThermoFisher Scientific®, UK). The DNA from the regolith setup were extracted on 0^th^ day, 7^th^ day, 14^th^ day and 21^st^ day and stored at -20 °C. Out of the 30 µl of volume containing the eluted DNA, 4–5 µl were used up for quantification by fluorometer (Qubit) and the remaining solution was used for library preparation for sequencing. If needed, one can skip the quantification and directly allow the nanopore device to detect any DNA that could be present. Figure 2 shows a schematic representation of the soil growth and DNA extraction, quantification, and sequencing procedure, applied in the clean room environment.

### Nanopore sequencing

#### Library preparation

The library for nanopore sequencing of all the samples was prepared using Ligation Sequencing Kit V14 (SQK-LSK114, Oxford Nanopore Technologies), which is compatible with their new chemistry R10.4.1 flongle (FLO-FLG114) flow cells. The gDNA for the nanopore library was prepared using nuclease-free water and the low concentration of the DNA to be tested was directly pipetted from the previously diluted samples, into DNA LoBind sterile eppendorf tubes (Eppendorf, UK). The whole amount of DNA (either from *E. coli, S. cerevisiae,* the mock community DNA, or obtained from each MMS-2 extraction), was diluted in 25 µl for library preparation. The preparation steps were followed according to the manufacturer’s instructions, except the volume for all the steps was halved as the samples were sequenced using a Flongle Flow Cell (R10.4.1) with a minimum of 50 or above active nanopores. As a negative control, a library with just nuclease-free water was prepared strictly following the protocol with all steps included. ONT’s Flongle Flow Cells require 125 µl priming mix and 30 µl gDNA library. It is important to note that the operator’s pipetting technique will considerably impact the accuracy while handling such low concentrations and may affect the result. At every library preparation stage, the specified reagents were transferred in an eppendorf tube before adding the sample containing gDNA, to avoid any losses due to pipetting.

#### Sequence analysis

The bases were called for 20–24 h using MinKNOW GUI software (ONT, V5.3.6). The passed Fastq files were analysed using EPI2ME Desktop Agent software (ONT, V3.5.7) with What’s In My Pot (WIMP) workflow that can produce a real-time taxonomic classification. WIMP is a workflow that classifies each sequence in the MinION™ FASTQ file uploaded by the Desktop Agent™^35^. The passed reads were filtered with a mean q-score (minQ) of 10 and for reads above this threshold, the Centrifuge classification engine assigned each sequence to a taxon based on the NCBI RefSeq database. The Centrifuge classification results were then filtered and aggregated to calculate, and report counts of reads at the species rank^36^.

#### Quality score threshold

For species identification, a certain quality threshold (minQ) must be set to reference the reads (those above the minQ score) against previously sequenced organisms. Using the Log10 quality score means that a quality score of 10 represents a 1 in 10 chance of an incorrect base call (a base call accuracy of 90%), whereas a quality score of 20 represents a 1 in 100 chance of an incorrect call^37^. The quality score output by ONT base callers is based on the outputs of the neural networks used to produce the base call. In our experiments the quality control (QC) score was set to 10. However, note that for life detection, also base-pair fragments identified with lower reliability (named as “fails” in the MinKNOW report) are useful, as they still represent reads of a polymer of base pairs (which may be less reliable for exact species identification if there is some chance of incorrect base call or below 200 bp).

## Results

### Single taxa lowest detection limit with MinION

In this work we successfully detected *E. coli* and *S. cerevisiae* DNA even at the level of 10 pg. Sequencing 10 pg of gDNA yielded 25 kb (average sequence length 7 kb) and 70 kb (average sequence length 9 kb) that were identified as *E. coli* and *S. cerevisiae,* respectively. For each gDNA sample, there were three technical libraries and three sequencing replicates (results are shown in Table 2). All the samples produced QC passed reads of 4 to 6. Nevertheless, the MinKNOW run report for replicate one, *E. coli* and *S. cerevisiae* produced a total of 224 and 337 reads respectively (including pass and fail reads) of which, ~ 20 k bases and ~ 60 k bases passed the minQ score. One of the replicates from yeast gDNA samples, revealed one read of other cross-contaminants like *E. coli* and *Paenibacillus Sonchi* (not shown) indicating the instrument’s environmental sensitivity. The negative control and the test samples detected traces of human DNA. The negative control with just nuclease-free water revealed human contamination (~ 4 kb) in an equivalent amount to the one detected in the samples with *E. coli* and *S. cerevisiae,* suggesting that there is possible human contamination despite working in ISO 5 clean room in all experiments at the level of pg that may either come from operator, reagents, instruments, and the ambient air.

**Table 2:** Replicates (R) of the Nanopore reads for each sample as per the MinKNOW report and EPI2ME WIMP classification.

Sample type	Total (pass and fail) reads			% reads used for species identification			Species	Qscore passed Nanopore reads		
	R1	R2	R3	R1	R2	R3		R1	R2	R3
10 pg of *E. coli* lenticule discs extract	224	73	313	2.7	5.5	0.3	*Escherichia coli*	4	3	1
							*Homo sapiens*	2	1	0
10 pg of YSC-2 yeast extract	337	37	190	1.5	13.5	1.1	*Saccharomyces cerevisiae*	4	4	2
							*Homo sapiens*	1	1	0
Negative control (Nuclease-free water)	180	72	269	1.1	6.9	1.1	*Homo sapiens*	2	5	3

It is important to note that any positive detection must be calibrated against the negative control reads and data. This calibration helps to avoid false positives when low biomass is used, especially for life detection on Mars.

### Detection of 2 pg of input DNA with MinION

To further verify the lowest detection limit, we diluted the *E. coli* and *S. cerevisiae* to 10 pg/µl which is Qubit’s lowest quantification limit, and prepared the library for two independent experiments, with only one-fifth of that preparation, i.e., 2 pg of gDNA of each type. MinION detected no reads in any of these preparations, suggesting that there may be a lower limit threshold for the hardware or software to operate and distinguish the signal from background noise. To overcome this threshold, we prepared two new experiments: 1) 2 pg of *S. cerevisiae* and 10 pg of *E. coli* and 2) 2 pg of *E. coli* and 10 pg of *S. cerevisiae* (see Table 3). We ran two replicates per sample type and the MinION sequencer successfully detected the microbial taxa at 2 pg in both experiments. This is the lowest limit detected so far.

**Table 3:** Two replicates of nanopore reads of 2 pg samples as per the MinKNOW report and EPI2ME WIMP classification.

Sample type	Total reads (pass and fail)		Species	Qscore passed reads	
	R1	R2		R1	R2
10 pg of *E. coli* + 2 pg of *S. cerevisiae*	411	1180	*Escherichia coli*	2	120
			*Saccharomyces cerevisiae*	2	17
			*Homo sapiens*	1	7
10 pg of *S. cerevisiae* + 2 pg of *E. coli*	2110	4700	*Saccharomyces cerevisiae*	243	263
			*Escherichia coli*	88	78
			*Homo sapiens*	19	53

### Community DNA lowest detection limit with MinION

**Figure 3:**
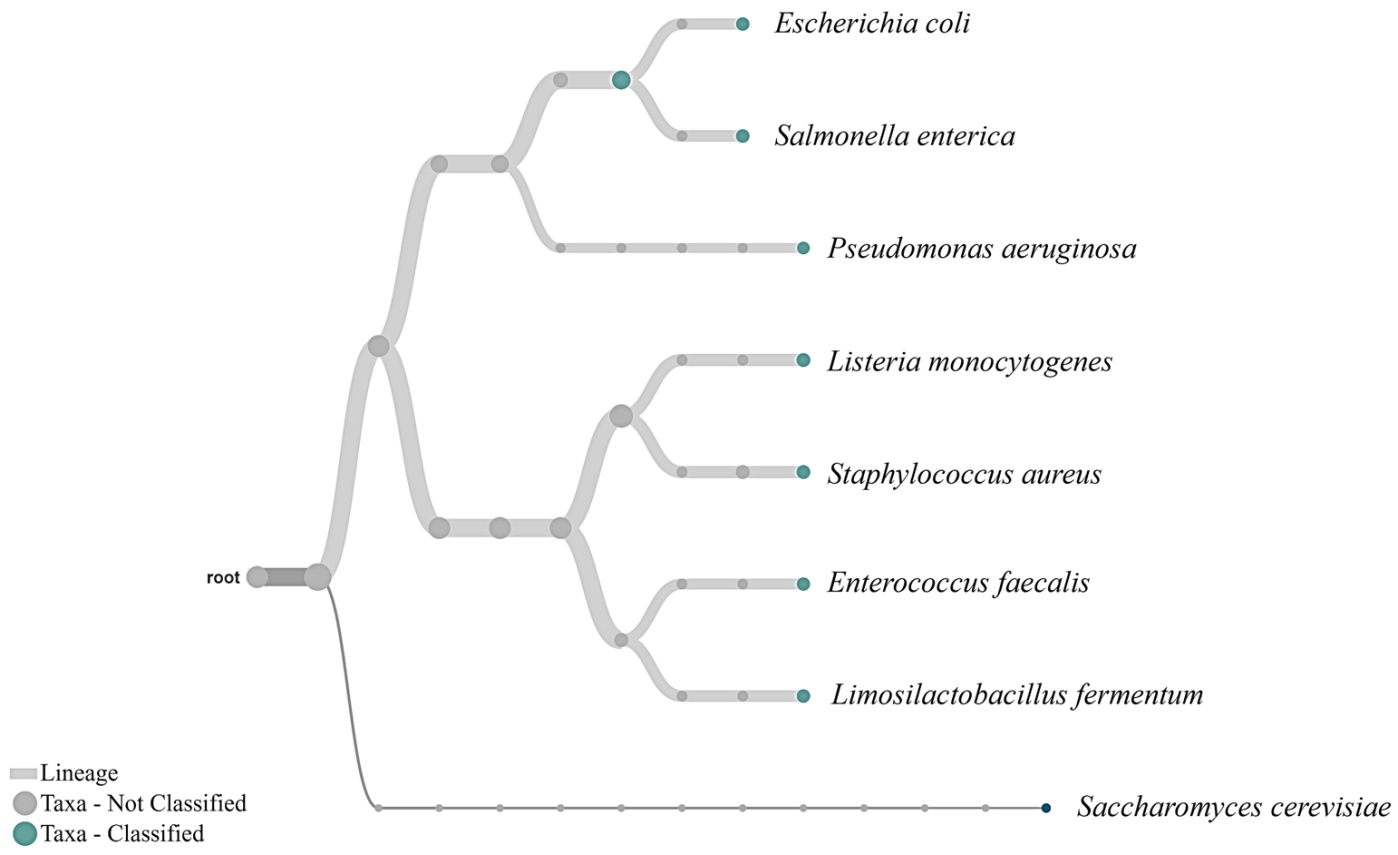
Phylogram showing the read classification based on the NCBI taxonomic classification obtained from sequencing 100 pg of the standard mock community DNA. EPI2ME, WIMP analysis with a minimum abundance cut-off of 1%, characterised eight of the ten species from the community including *Saccharomyces cerevisiae* which is only ≤ 2 pg of DNA within the prepared sample. The image is a representation of one technical replicate out of the two. See supplementary Fig. 1 for another replicate.

After confirming the lowest quantity of gDNA required to sequence and detect a single taxon, we tested the limits of detection with a mock community representing a mix of different taxa (Table 1). With the Zymobiomics mock community DNA sample, we tested concentrations of 1000 pg, 750 pg, 500 pg, 250 pg, 100 pg, 50 pg and 10 pg. Nanopore sequence analysis of samples with low concentrations of 10 pg or 50 pg resulted in the detection of two to six bacterium taxa. At these low levels of DNA, the results are sensitive to the pipetting process, as each case may have randomly selected one or another type of analyte within the extremely diluted solution. A total DNA mass of 100 pg (0.1 ng) was the lowest amount that identified 80% of the taxa i.e., eight out of ten organisms in the mock community DNA sample. This detection limit is one order of magnitude lower than ONT’s specifications for studies without amplification (1 ng). As highlighted in Table 1, for a total amount of 100 pg, eight organisms are on an average expected to be represented with only 12 pg, while two organisms are represented on an average with 2 pg which is consistent with the detection limit of gDNA from single organism, described above. It should be noted that gDNA proportion, as described in Table 1, are based on a relative proportion and not an absolute value of each organism’s gDNA that is present in the initial template DNA. For one technical replicate, a total of 862 reads were generated of which, 450 were passed quality reads, among which all the bacterial reads were between 50–60, and yeast was 4–6. We also detected two reads classified as *Homo sapiens* which, as in the case above, are indicative of the cross-contamination produced by the kit reagents, or the operator itself. The ability to sequence and detect *Saccharomyces cerevisiae* which was only 2% of the total gDNA in the mock standard, confirms the possibility of detecting down to 2 pg of DNA in the library that is used for MinION nanopore sequencing. Read classification output for a 100 pg sequencing run is represented as a phylogram in Fig. 3, and the other replicates of 100 pg sequencing are shown in supplementary Fig. 1.

### Extractions from MMS-2 under different incubation conditions

**Figure 4:**
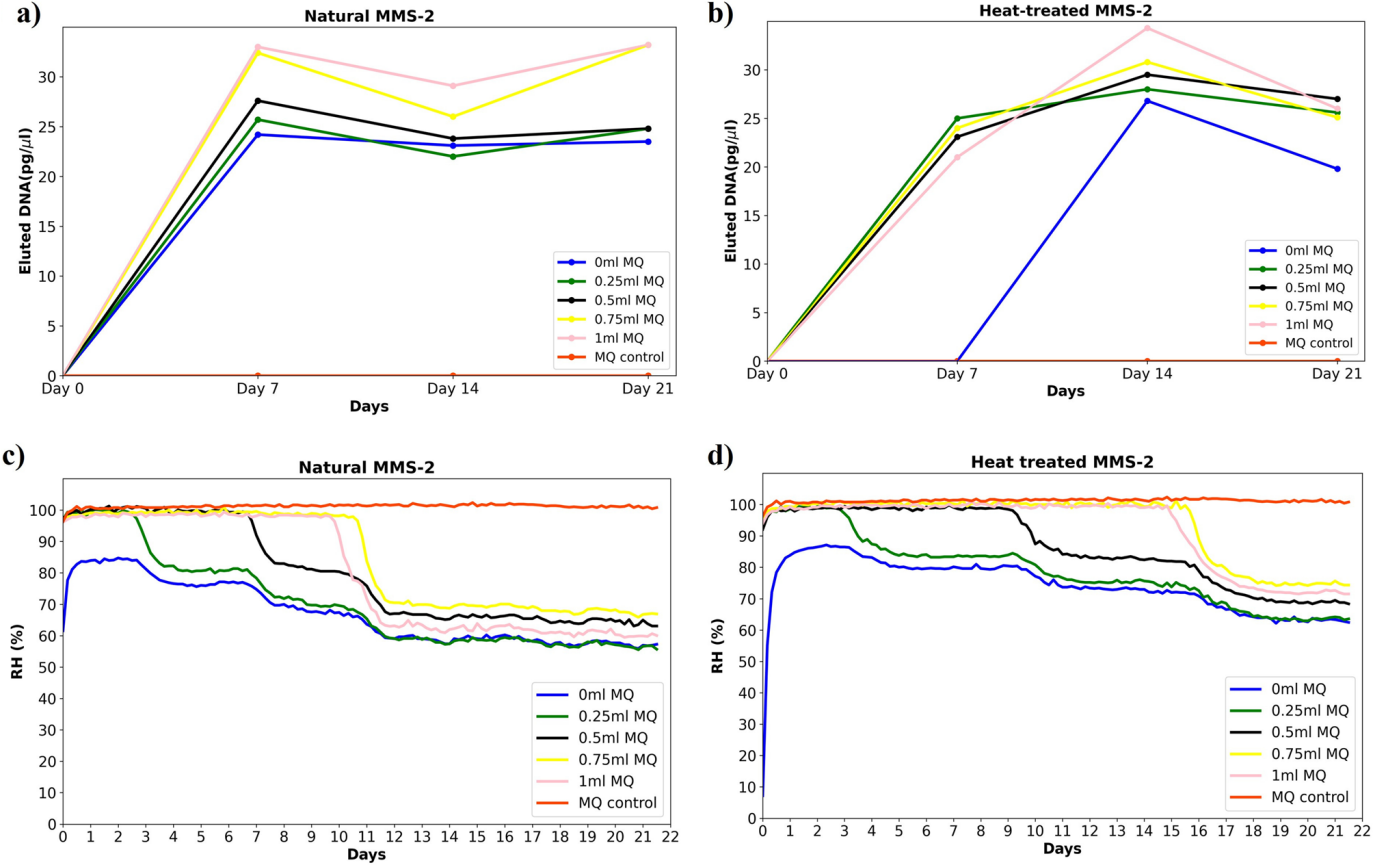
Martian analogue soil cultivation experiments at 30 °C. Line chart showing DNA yields from 500 mg of MMS-2 for both (**a**) natural and (**b**) heat-treated soils at different intervals. The plot averages three replicates with standard deviation ranging between 0 and 0.021. Relative humidity (RH %) evolution in the experimental setup for (**c**) natural and (**d**) heat-treated MMS-2 soils, for all experimental conditions in the well plate. These parameters were logged every four hours for 21 days with an RH resolution of 0.04%. The incubator used for the cultivation is a forced convection hot air oven working in atmospheric conditions.

**Figure 5:**
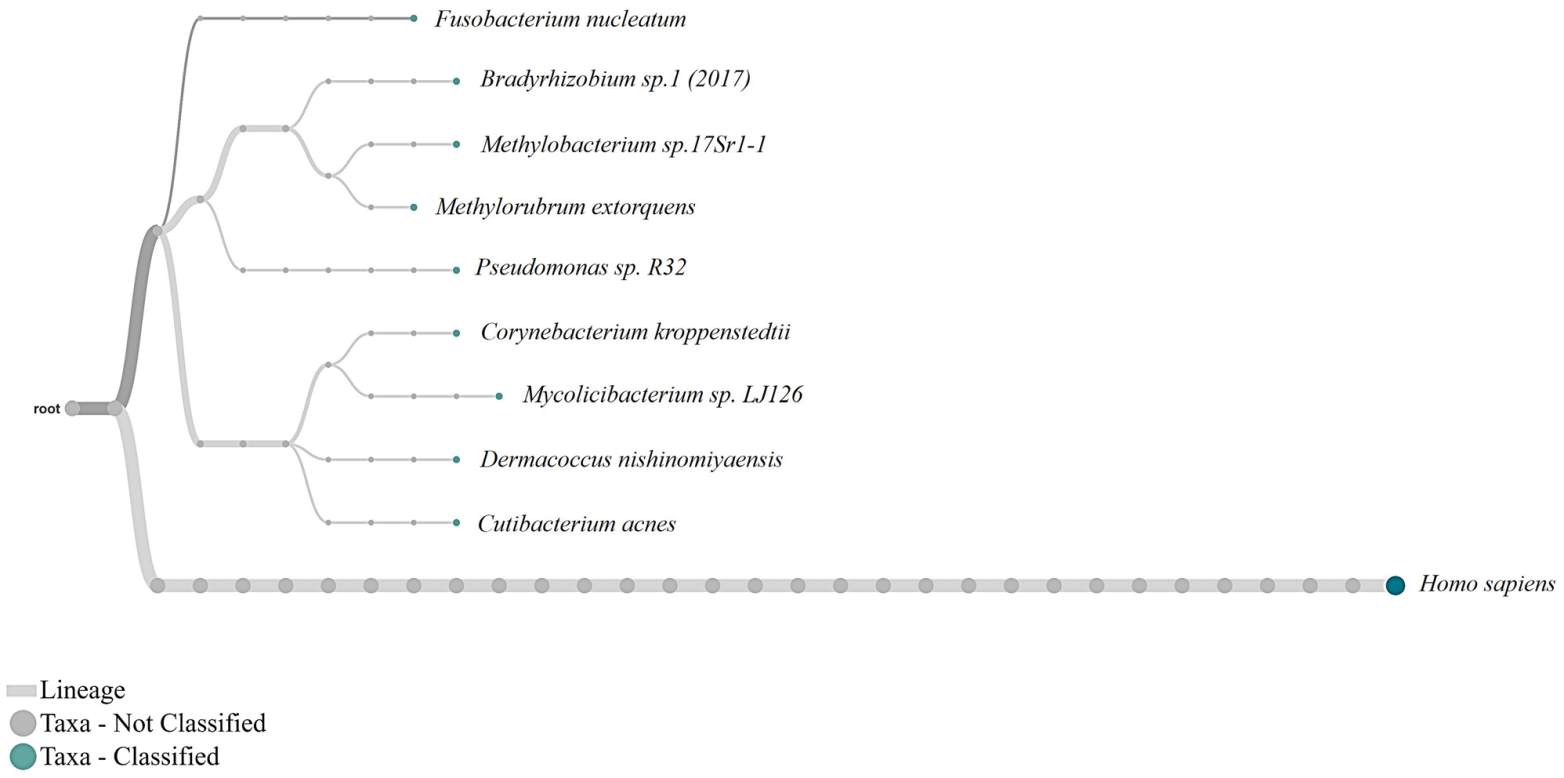
WIMP NCBI taxonomic classification obtained from sequencing ~ 600 pg of DNA extracted from natural dry MMS-2 incubated for 7 days at 30 °C in atmospheric vapour (RH 70%). With a minimum abundance cut-off of 1%, WIMP analysis characterised 41 reads out of 52 passed reads as soil microbes and airborne pathogens or contaminants. The minimum qualifying score for analysis was 10.

In order to mimic a natural low biomass sample, the regolith growth experiments were setup with different amounts of water, including a dry-soil experiment exposed only to atmospheric humidity, using both untreated and heat-treated MMS-2. The first DNA extraction for both types of MMS-2, occurred on day 0, right at the start of the incubation experiment, showing no detectable DNA by fluorometric analysis. For the rest of the extractions, the total DNA obtained per 500 mg of soil for each experiment was in the range of 600 pg to 1000 pg as measured by fluorometric quantification. The obtained DNA yield is presented as a probable growth curve in Fig. 4a,b, wherein we observed that the maximum amount of DNA was extracted on day 7 and day 14 from natural and heat-treated MMS-2 conditions, respectively. Although since the quantity of obtained DNA in all cases is at the level of pg, and each week’s experiment is a different replicate, the difference in DNA yield is not significant. However, the transition from zero to 600 pg to 1000 pg of DNA could suggest microbial growth within the soil. The microbes that grow within the regolith could be indigenous (or at most have been transferred through the air or sample crushing procedures through the manipulations prior to the experiment). For each condition, the water activity was monitored using iButtons with a resolution of 0.04% relative humidity (RH). The temperature data (resolution of 0.0625 °C), which was also logged, was within the range of 29.5 °C and 30.5 °C, as expected for the incubator. The initial moisture content of the natural MMS-2 soil was recorded for an hour, and the result suggested a water activity average of 0.35 (35% RH). For the heat-treated MMS-2 soil, the initial regolith water activity was reduced to 0.07 (7% RH). Once the regolith is placed within the wells, nearby to the other experiments with added liquid water, and the control well with nuclease-free water, the water activity of the dry soil experiments rises within 48 h typically to 0.8 (80% RH) due to the exposure to the water in the headspace gas. The heat-treated soil took a few hours more to rehydrate. The RH readings for untreated and heat-treated soils were plotted as curves, as shown in Fig. 4c,d. From the data, we can clearly observe how water evaporates from the wet experiments and vapour is redistributed through the headspace of each well, changing the water activity of each experimental setup. We next characterise the microbial communities that grow in dry soils (0 ml MQ) conditions by sequencing the extracted DNA (Fig. 5 and Table 4).

**Table 4:** Summary of the WIMP classified microbes with abundance > 0.5% and the passed read counts of dry MMS-2 from different incubation intervals.

Sample	Natural dry MMS-2							
	Day 0	Reads	Day 7	Reads	Day 14	Reads	Day 21	Reads
(a) Natural dry MMS-2								
Species (Passed and failed reads count)	*Pseudomonas sp. J380*	1	*Fusobacterium nucleatum*	1	*Escherichia coli*	2	*Cytophaga hutchinsonii*	1
	*Homo sapiens*	5	*Bradyrhizobium sp. 1(2017)*	1	*Listeria monocytogenes*	2	*Homo sapiens*	3
	Unclassified	1	*Methylobacterium sp. 17Sr1*	1	*Salmonella enterica*	1	Unclassified	2
	Failed reads	410	*Methylorubrum extorquens*	1	*Enterobacteriaceae*	1	Failed reads	204
			*Pseudomonas sp. R321*	1	*Homo sapiens*	2		
			*Corynebacterium kroppenstedtii*	1	Unclassified	2		
			*Mycolicibacterium sp. LJ126*	1	Failed reads	156		
			*Dermacoccus nishinomiyaensis*	1				
			*Cutibacterium acnes*	1				
			*Homo sapiens*	31				
			Unclassified	11				
			Failed reads	1669				
Total		417		1720		166		210
% of passed reads		1.7%		2.9%		6.0%		2.9%

Sample	Heat -treated MMS-2							
	Day 0	Reads	Day 7	Reads	Day 14	Reads	Day 21	Reads
(b) Heat treated dry MMS-2								
Species (Passed and failed read counts)	*Pseudomonas sp. J380*	1	*Homo sapiens*	7	*Candidatus nitrosacidococcus tergens*	1	*Novosphingobium ginsenosidimutans*	1
	*Homo sapiens*	1	Unclassified	2	*Micrococcus luteus*	1	*Homo sapiens*	5
	Unclassified	0	Failed reads	135	*Azotobacter vinelandii*	1	Unclassified	2
	Failed reads	107			*Homo sapiens*	8	Failed reads	948
					Failed reads	457		
Total		109		144		468		956
% of passed reads		1.8%		6.3%		2.4%		1.0%

### MinION nanopore sequencing of DNA extracted from dry MMS-2

Nanopore sequencing using the MinION platform can identify DNA at lower concentrations than the fluorometer (Qubit 4.0, which does not detect below 10 pg). To verify the MinION sequencer’s low detectability with a natural low biomass sample, we sequenced the DNA extracted from days 0, 7, 14 and 21 of both natural and heat-treated dry MMS-2. For natural and heat-treated conditions, the analysis of DNA extracted from the soils at day 0 generated 417 and 109 reads, respectively, out of which < 10 reads passed the Qscore (10). The passed reads were classified by WIMP as *Homo sapiens* and *Pseudomonas* (Table 4), which may have been added as contamination during the library preparation or exist within the soil due to previous contamination. Sequencing just ~ 600 pg of DNA obtained from day 7 natural MMS-2 extraction without any amplification generated a total of 1720 reads, out of which 52 QC passed reads were classified as typical soil inhabitants such as *Bradyrhizobium, Methylobacterium, Methylorubrum* and other airborne microbes.

DNA from day 7 of heat-treated dry MMS experiments also showed no signs of detectable DNA by fluorometer, but the sequencing resulted in 7 reads of *Homo sapiens,* indicating low contamination. DNA extractions from 14 days of incubation of heat-treated dry MMS-2 had 20 QC passed reads that were classified as nitrogen fixing bacteria such as *Micrococcus luteus* and *Azotobacter vinelandii*. The remaining characterisation of dry MMS for natural and heat-treated MMS-2 has been summarised in Table 4. Depending on the ecosystem, the bioinformatics pipeline for microbial classification, taxonomic database and genomic assembly can be modified to identify as many reads as possible. This model characterisation of a natural and heat-treated MMS-2 soil at picogram level without any amplification is a significant advancement for the study of Martian or terrestrial samples with extremely low biomass.

## Discussion

Oxford Nanopore Technologies (ONT) recommends a minimum of 1 ng to 400 ng of DNA with and without amplification respectively for successful sequencing outputs. Other Next Generation Sequencing (NGS) platforms, such as Illumina and PacBio, require high-quality DNA and their latest ultra-low input sequencing can be performed with a minimum of 1 ng and 5 ng respectively^38^. Though the other NGS technologies have lesser error rates than Nanopore, the need for an amplification step for low DNA concentration can mis-amplify the DNA with the existing primer sets, opening doors for contamination and false positive reports^39^. Therefore, our study aimed to explore the lowest limits of nanopore sequencing without amplification. With the release of new flow cell chemistry (R10.4.1) and its ligation sequencing kit (V14) with improved high consensus accuracy by ONT^40^, we were able to test this product for all our low detection experiments using a portable MinION sequencer. Our study shows that MinION sequencer can unequivocally detect and characterise species with as little as 2 pg of DNA with just 50 active nanopores. Previous works have explored this possibility of characterisation with 10 pg of high-biomass DNA, but with a PCR amplification step^41^. Hence, our results present a significant advancement in sensitivity, particularly applicable to investigating low biomass samples and novel exploitation of the sequencer pushing its detection limits to 1/500,000th without amplification. However, our study also highlights that at the level of 2 pg, cross-contamination of DNA from humans, reagents, and ambient air could be significant, suggesting the need of a higher class of clean room and highly sterilised kit reagents for testing at picogram level. Notice that the analysis of nuclease-free water already gives 72 to 269 reads of DNA; this defines a systematic background level of possible contamination for all experiments. Therefore, control samples with no DNA template are vital while sequencing at the picogram level to identify false positives occurring from the above sources, even in a clean room. The negative controls specifically help calibrate the positive reads against it and mitigate misclassification.

The DNA sensitivity limit with the Flongle Flow Cell of ONT depends on the purification and concentration process from the original sample, either solid or liquid, to the final analyte injected into it. For instance, in our experiments with the MMS-2 soil, the Flongle Flow Cell was able to characterise the microbiome with as little as 600 pg of DNA extracted from 500 mg of soil sample. It should be noted that the actual life detection potential is even much higher, as the fragments discarded due to the potentially lower reliability of the base-pair identification in the MinION sequencing and assigning procedure, are also unique indicators of the presence of a biofunctional polymer. In all studied cases, there were typically between 100 and 1700 DNA reads, per 500 mg of soil. Of the total amount of DNA (failed and passed) reads only about 1 to 6% (passed reads) have been used for species identification, see Table 4. This result suggests that DNA detection based on reads of base-pair polymers can be sensitive to two orders of magnitude less than initial DNA concentration or DNA mass.

Our study with the MMS-2 Mars soil simulant, which is produced by crushing basaltic rocks to the size of Martian regolith, shows one way to characterise Mars returned samples or any low biomass terrestrial environments without addition of nutrients. After 7 days of exposure to environmental conditions with water activity a_w_ greater than 0.6 (with atmospheric water or liquid water), all soil samples showed an increase in DNA concentration, leading to the accumulation of 600 to 1000 pg of DNA, per 500 mg of soil (starting from no detectable DNA). On Earth, the lowest reported limit of water activity that enables cell division appears to be ∼ 0.6^42^. Interestingly, in the case of regolith samples that were pre-dried at 125 °C in the hot air oven for 10 h, the dry MMS-2 condition did not result in any DNA accumulation, until two weeks later. However, if liquid water is added at the onset of the experiment, the growth seems to be similar to the ones of the untreated regolith. We detected reads related to soil organisms such as *Bradyrhizobium*, which fix nitrogen, and *Methylobacterium sp. 17Sr1*, a facultative methylotrophic bacterium that has shown to be resistant to gamma and UV radiation^43^, *Cytophaga hutchinsonii*, a common cellulolytic soil^44^, *Candidatus Nitrosacidococcus tergens*, a gammaproteobacterial bacterium that can oxidate ammonia at pH 2.5^45^, some of which are also associated with kit reagents contamination. Previous studies have detected bacterial contamination from ultraclean reagents despite working in ultraclean facilities^46, 47^. Another study also detected bacterial DNA contaminants such as *Actinomyces, Pseudomonas spp, Enterococcus* etc. from DNA extraction kit reagents and PCR reagents in a low biomass environment, some of which were common to our dry MMS-2 characterisation^48^. We also found similar kitome microbiome such as *Pseudomonas, Actinomycetes, Shigella, Enterobacteriaceae* in our 2 pg test samples as well as Martian regolith samples. The picogram detection level of the MinION nanopore technology together with the procedure presented here, may be of interest for the future Mars Sample Return program, and for the life research and planetary protection studies that will be implemented through the Sample Safety Assessment Framework^24^ to be applied in the Biosafety Level 4 clean room. Once on Earth, one of the main investigations to be implemented with the samples will be to assess the presence of life as we know it and protect Earth from biological hazards.

Our work within the ISO5 clean room environment, and the ubiquitous presence of cross-contamination, suggests that the analysis of potential life forms within the Martian rocks on Earth will require to implement rigorous methods to avoid the cross-contamination of the biospheres from two terrestrial, habitable planets, namely Earth and Mars. This implementation will require working in containment within a Biosafety Level 4 facility and constructing a complete genetic inventory of the environment where the samples will be stored and manipulated. Also, it is of utmost importance to include negative controls and standards while sequencing any low biomass environment. These are required prerequisites to investigate if any extant or preserved (dormant) life form has the potential to replicate.

## Conclusion

This study has demonstrated the potential of MinION nanopore technology to: (1) Detect DNA strands from a monoculture with an input as little as 10 pg and mixed culture with 2 pg. (2) Characterise with an ISO5 clean room environment, the possible ambient Earth contamination even with nuclease-free water as DNA input, and (3) Identify microorganisms with extremely low biomasses, down to 1 ppm (i.e., 1 × 10^–6^) of the rock sample, and detect nucleotide base-pair polymers in concentrations of 1 × 10^–8^ of the rock sample.

After DNA extraction in a liquid solvent, its quantification with a fluorometer can be used as a parameter to monitor microbial growth. The portable MinION sequencer is particularly useful for taxonomic identification at or below the species level, and allows the distinction of contamination sources, such as human DNA. We suggest that a Martian soil, without any added nutrients, could support the growth or revival of microorganisms by mere exposure to atmospheric moisture with water activity below 0.85. These conditions are met at the surface of present-day Mars, although at much lower temperatures. In future studies, this method could also be used to investigate the growth of soil microorganisms under other extreme environmental conditions that are representative of the present-day near-surface Martian conditions such as exposure to salts and oxidating radicals, low temperatures, and exposure to ionising radiation from cosmic and solar radiation.

## Supplementary Information

Supplementary Information.

## Data Availability

All sequencing data in this study have been generated using ONT’s MinKNOW software. The data was analysed using their EPI2ME WIMP software using the Fastq pass files and are stored in ONT’s cloud. All the sequencing files (Fast5 and Fastq) are available on Zenodo with an open access, 10.5281/zenodo.8208597. Editors and readers can contact the corresponding author for accessing any other datasets.
