# Author Correction: DNA sequencing at the picogram level to investigate life on Mars and Earth

**DOI:** 10.1038/s41598-025-98000-4

**Published:** 2025-04-23

**Authors:** Jyothi Basapathi Raghavendra, Maria‑Paz Zorzano, Deepak Kumaresan, Javier Martin‑Torres

**Affiliations:** 1 Department of Planetary Sciences, School of Geosciences, University of Aberdeen, Meston Building, Aberdeen, AB24 3UE Scotland; 2 Centro de Astrobiología (CAB), CSIC-INTA, 28850 Torrejón de Ardoz, Madrid, Spain; 3 School of Biological Sciences, Queen’s University Belfast (QUB), Belfast, BT9 5DL Northern Ireland; 4 Instituto Andaluz de Ciencias de La Tierra (CSIC-UGR), 18100 Granada, Spain

Correction to: *Scientific Reports* 10.1038/s41598-023-42170-6, published online 15 September 2023

The original version of this Article contained errors in Figure 4a and b, where the data points were interchanged. The negative control data points were therefore incorrectly represented as these were stated as non-zero, which contradicted the quantifications of the other conditions.

**Fig. 4:**
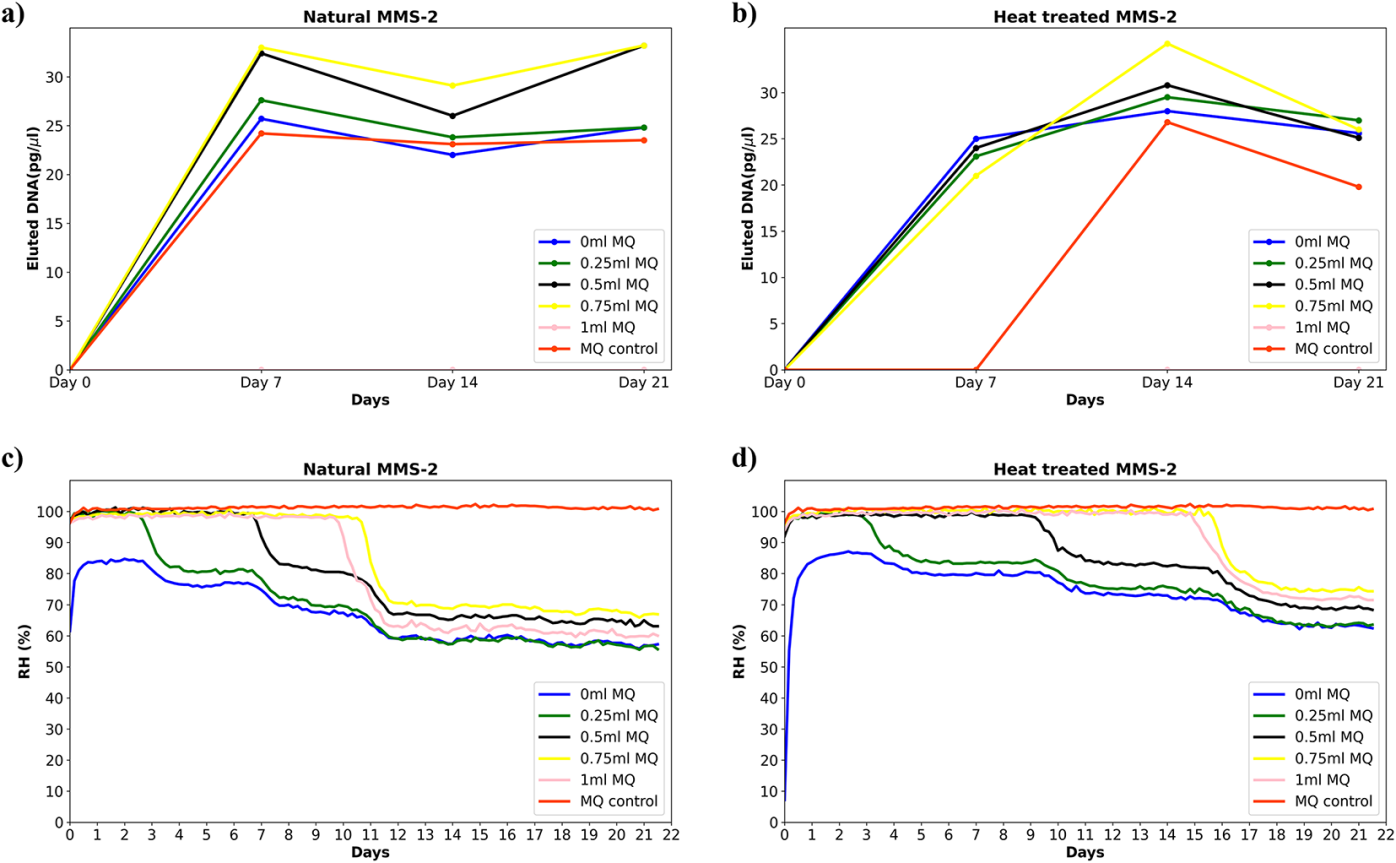
Martian analogue soil cultivation experiments at 30 °C. Line chart showing DNA yields from 500 mg of MMS-2 for both (**a**) natural and (**b**) heat-treated soils at different intervals. The plot averages three replicates with standard deviation ranging between 0 and 0.021. Relative humidity (RH %) evolution in the experimental setup for (**c**) natural and (**d**) heat-treated MMS-2 soils, for all experimental conditions in the well plate. These parameters were logged every four hours for 21 days with an RH resolution of 0.04%. The incubator used for the cultivation is a forced convection hot air oven working in atmospheric conditions.

The original Figure 4 and accompanying legend appear below.

The original Article has been corrected.

