# Supplementary material for: DNA sequencing at the picogram level to investigate life on Mars and Earth
Source: Sci Rep. 2023 Sep 15;13:15277. doi: 10.1038/s41598-023-42170-6 (PMC10504319; doi:10.1038/s41598-023-42170-6)
Supplement: Supplementary file 1 — Supplementary Information. [file 41598_2023_42170_MOESM1_ESM.docx]

**DNA sequencing at the picogram level to investigate life on Mars and Earth**

**Jyothi Basapathi Raghavendra^1^*, Maria-Paz Zorzano^2^, Deepak Kumaresan^3^ and Javier Martin-Torres^1,4^**

**Supporting Information**

**Replicate of community DNA lowest detection limit:**

**
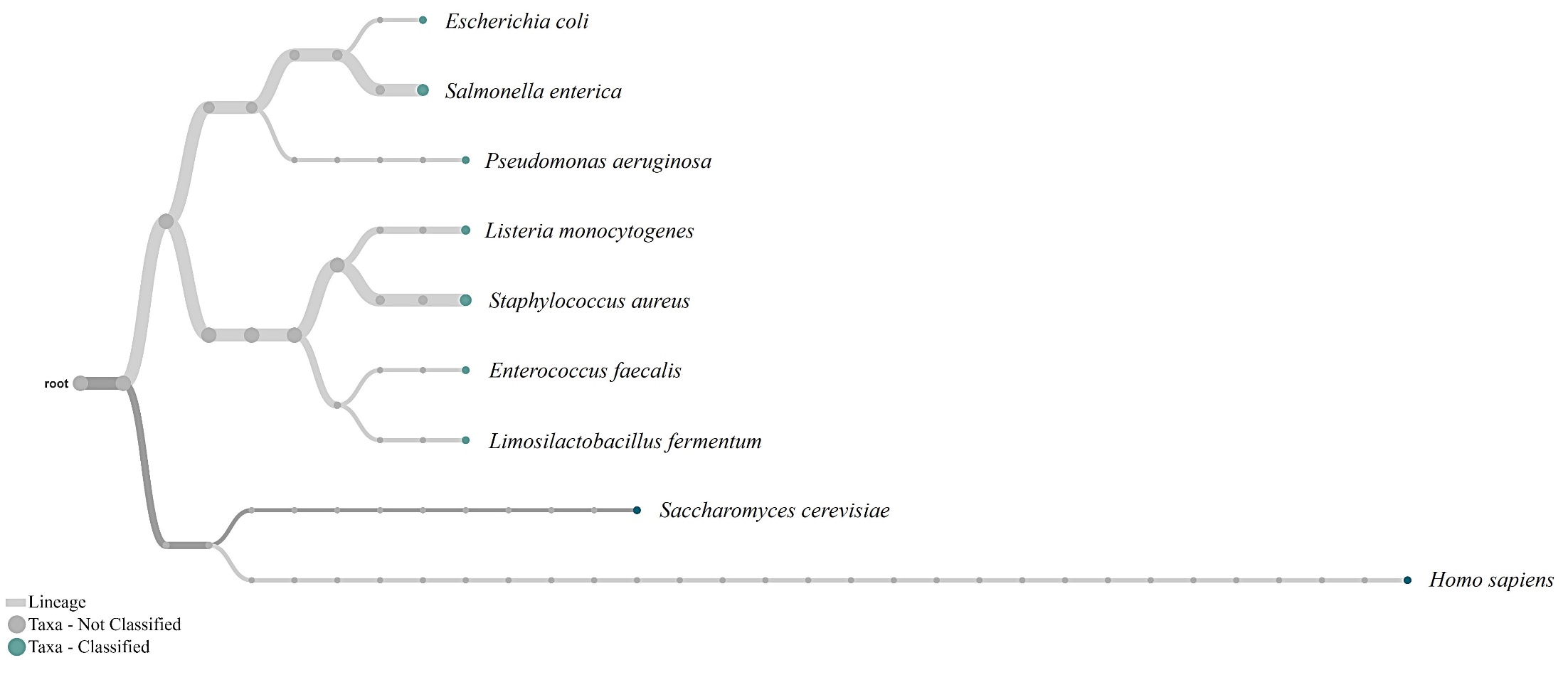
**

**Figure 1:** The NCBI taxonomic classification obtained from sequencing 100 pg of the standard mock community DNA. EPI2ME, WIMP analysis with a minimum abundance cut-off of 1%, characterised eight of the ten species from the community including *Saccharomyces cerevisiae* which is only ≤ 2 pg of DNA within the prepared sample similar to its replicate. With 373 reads generated, 19 of them passed the Qscore and were classified as shown above. Out of these 17 reads were prokaryotic and 2 reads were eukaryotic (One yeast and one *Homo sapiens*).

**Cleanroom monitorisation:**

To examine the microbial concentration inside the clean room, 1000L of air at 50L/min was sampled using a Sartorius air sampler with a sterile gelatine filter (Sartorius, 17528-80) that captures the microbes in the ambient air. The sampled gelatine was then aseptically transferred to a sterile ready-to-use R2A plates (SLS, UK). As controls, a sterile unsampled filter and outdoor sampled (1000 L of air) filters were cultivated on the plate in an [incubator](https://www.sciencedirect.com/topics/engineering/incubator) at 30 °C, for 72 hours. As per the ECSS-Q-ST-70-55C standard, an area of 0.0025 m^2^ was swabbed using a sterile Nylon swab (FLOQSwabs® from Copan) and dipped in an Eppendorf tube containing 500µl of sterile 1X PBS. The swabbing was performed at an angle of 30° to the surface and repeated twice, changing the direction of the swabbing motion by 90°. As controls, a swab without any material contact and an outdoor swab were performed. After shaking the swab in PBS, the respective samples were pipetted on a sterile ready to use R2A plates (SLS, UK) and gently spread using a L-shaped sterile spreader and incubated at 30 °C for 72 hours. The agar plates after incubation are seen in Figures 2a and 2b for air quality monitoring and swab assays, respectively. Both the above protocols were previously conducted by our team similarly and published the results^[49]^. Prior to the experimental setup inside the cleanroom, the working table, inside wall panels and equipment were sprayed with 70% isopropyl alcohol (RS components, UK) and Chemgene HLD_4_L (Medimark Scientific Ltd, UK). The space was thoroughly wiped using sterile cleanroom wipes (VWR, UK).


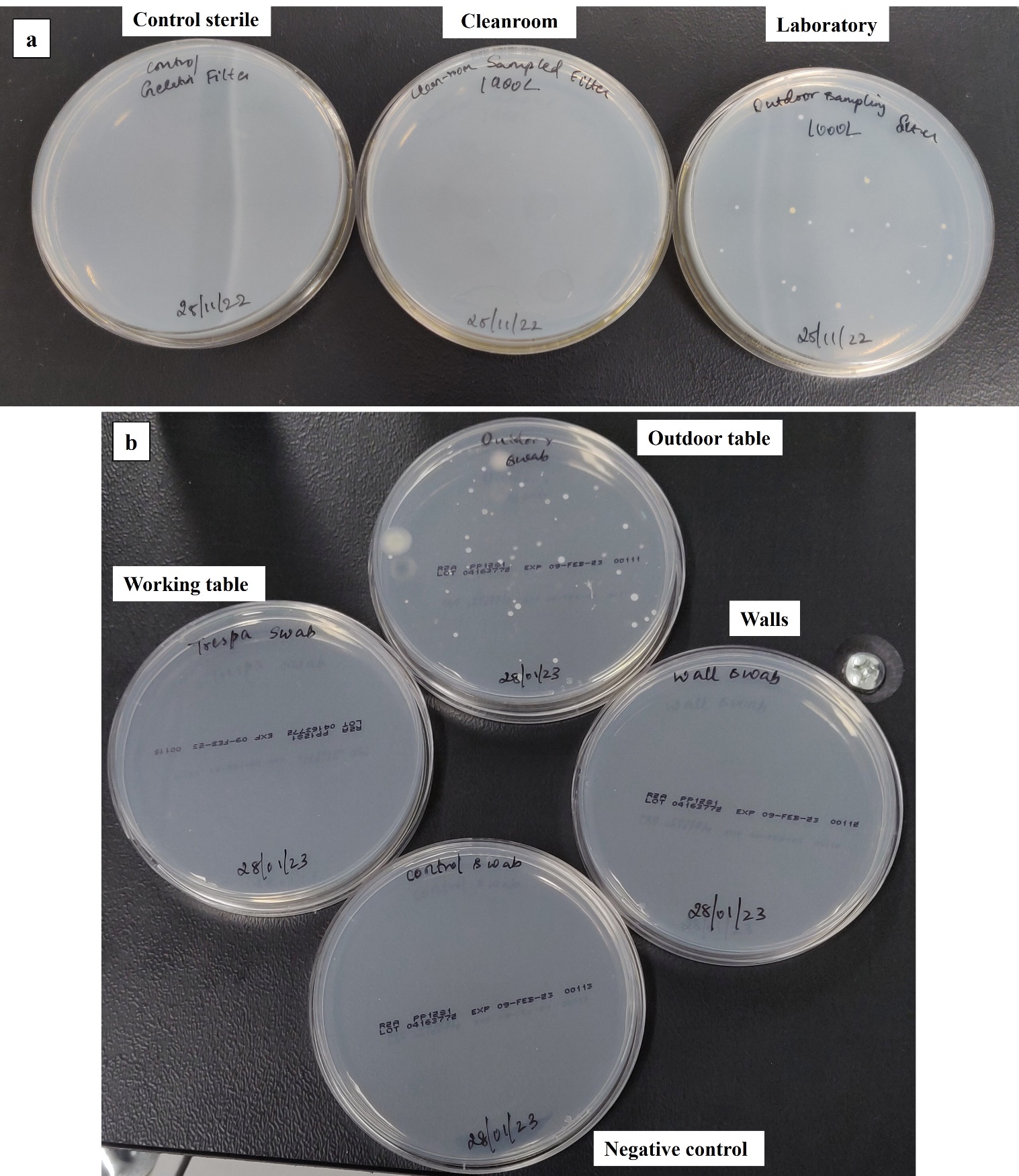


**Figure 2:** a) Microbial colonies formed after 72h of incubation at 30 °C from the 1000 L of air sampled inside and outside the cleanroom b) Swab assay to examine the microbial contamination level inside the clean room. ~500 µl of swabs were spread on the plates and incubated at 30 °C for 72 hours. The plates containing clean room swabs plates were free of any colony or microbial growth. Few microbial colonies grew on the R2A plate with a swab from the laboratory trespass (outside the clean room), validating our swab assay.
